# 3D Printed Model of Extrahepatic Biliary Ducts for Biliary Stent Testing

**DOI:** 10.3390/ma13214788

**Published:** 2020-10-27

**Authors:** Joanna Thomas, Sagar Patel, Leia Troop, Robyn Guru, Nicholas Faist, Brian J. Bellott, Bethany A. Esterlen

**Affiliations:** 1 Biomedical Engineering Department, Mercer University, Macon, GA 31207, USA; patel_SG@mercer.edu (S.P.); leia.danielle.troop@live.mercer.edu (L.T.); robyn.guru@live.mercer.edu (R.G.); Nicholas.ryan.faist@live.mercer.edu (N.F.); 2 Chemistry Department, Western Illinois University, Macomb, IL 61455, USA; b-bellott@wiu.edu (B.J.B.); Ba-esterlen@wiu.edu (B.A.E.)

**Keywords:** extrahepatic bile ducts, 3D printing, in vitro testing, mechanical properties, biliary stents

## Abstract

Several inflammatory conditions of the bile ducts cause strictures that prevent the drainage of bile into the gastrointestinal tract. Non-pharmacological treatments to re-establish bile flow include plastic or self-expanding metal stents (SEMs) that are inserted in the bile ducts during endoscopic retrograde cholangiopancreatography (ERCP) procedures. The focus of this study was to 3D print an anatomically accurate model of the extrahepatic bile ducts (EHBDs) with tissue-like mechanical properties to improve in vitro testing of stent prototypes. Following generation of an EHBD model via computer aided design (CAD), we tested the ability of Formlabs SLA 3D printers to precisely print the model with polymers selected based on the desired mechanical properties. We found the printers were reliable in printing the dimensionally accurate EHBD model with candidate polymers. Next, we evaluated the mechanical properties of Formlabs Elastic (FE), Flexible (FF), and Durable (FD) resins pre- and post-exposure to water, saline, or bile acid solution at 37 °C for up to one week. FE possessed the most bile duct-like mechanical properties based on its elastic moduli, percent elongations at break, and changes in mass under all liquid exposure conditions. EHBD models printed in FE sustained no functional damage during biliary stent deployment or when tube connectors were inserted, and provided a high level of visualization of deployed stents. These results demonstrate that our 3D printed EHBD model facilitates more realistic pre-clinical in vitro testing of biliary stent prototypes.

## 1. Introduction

**Figure 1:**
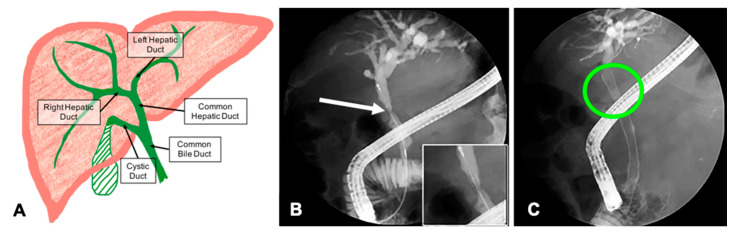
Hepatobiliary anatomy and cholestasis caused by a biliary stricture. (**A**) Anatomically, the extrahepatic ducts begin at the left and right hepatic ducts. They merge to form the common hepatic duct that connects to the gall bladder via the cystic duct. Bile drains into the small intestine through the common bile duct. (**B**) A significant stricture of the common hepatic duct (CHD) viewed during an endoscopic retrograde cholangiopancreatography (ERCP). The stent catheter has been threaded through the stricture. (**C**) A SEM stent was deployed to open the stricture and alleviate the cholestasis. (**B** and **C** modified with permission from [17]).

Bile is produced in the liver and drains through the bile ducts into the small intestine where it aids in digestion (Figure 1A). Cholestasis occurs when the bile ducts are inflamed or blocked, and bile is unable to drain out of the liver. Several conditions can cause cholestasis including gallstones, primary sclerosing cholangitis (PSC), and cholangiocarcinoma [1,2,3]. Treatment for acute or chronic cholestasis includes insertion of plastic or self-expanding metal stents (SEMs) into the extrahepatic bile ducts (EHBD) to re-establish bile flow to the intestines (Figure 1B,C).

Due to anatomical constraints, biomedical devices like biliary stents cannot be in vivo tested in small animal research models; these devices must be tested in porcine, ovine, or non-human primate models. Efficient and accurate in vitro testing of these devices during pre-clinical studies, in lieu of large animal testing, reduces the number of research animals that undergo procedures and/or are sacrificed to obtain the pre-clinical data required for submission to the FDA [4,5,6]. To date, in vitro studies of biliary stents have been performed in systems that do not mimic the anatomy and/or the mechanical properties of the EHBD [7,8,9,10].

The EHBDs are comprised of the common bile duct (CBD), cystic duct, common hepatic duct (CHD), left hepatic duct (LHD), and right hepatic duct (RHD). The duct walls, lined with cholangiocytes and formed by a thin layer of smooth muscle and connective tissue, serve as the conduit for bile between the liver and the small intestine. Ex vivo studies on porcine and human CBDs demonstrated the tissues are bi-axially elastic and relatively strong [11,12]. Therefore, a lab-generated, biomimetic EHBD model must be elastic, pliable, unaffected by exposure to bile at body temperature, dimensionally to-scale, and biocompatible. While tissue engineering of bile ducts is ever more feasible [13,14,15], the protocols can be costly to execute, difficult to reproduce, and time intensive, making them not yet a practical source of EHBD for pre-clinical biliary stent studies [16]. Alternatively, a growing number of polymers possessing a variety of mechanical properties are now commercially available for use in 3D printers. Herein, we describe our efforts to fabricate an anatomically accurate EHBD model with Formlabs 3D printers and polymers for in vitro biliary stent testing.

## 2. Materials and Methods

### 2.1. Computer Aided Design (CAD) and 3D Printing of the EHBD Model

We generated three CAD models of the EHBD with wall thicknesses of 0.5 mm, 0.75 mm, and 1 mm, based on average anatomical EHBD dimensions [18,19,20] in Autocad Fusion360 software. The models were then exported as .STL files. With Formlabs PreForm software, the .STL files were oriented and supported to facilitate printing in FE, FD, or FF on a Formlabs Form2 or Form3 SLA printer and saved as form files. The unique EHBD form files for each resin were uploaded to and printed with the designated resin on a Formlabs Form2 or Form3 printer. All the models were printed at 100 μm layer height; pre-programmed printing temperature was 31 °C for all three resins. After printing, the models underwent optimized post-processing (see Supporting Materials). In brief, they were washed (while still attached to the print bed) for 20–30 min in >95% isopropanol (IPA) in a Formlabs Wash Station. The EHBD models were then detached from the print bed, removed from the support trusses, and ultraviolet (UV) cured according to Formlabs guidelines for each resin (Form Cure Settings) in a Formlabs UV Cure Station. Following the UV cure cycle the models were promptly removed from the UV Cure Station.

### 2.2. Material Characterization of Elastic Polymers

A CAD model of an ASTM D-1708 micro-tensile sample was generated with Autocad Fusion360 software. The micro-tensile sample .STL file was imported into PreForm software and prepped for printing in FE as described above. Printed samples were removed from the print bed prior to washing for 30 min in >95% IPA. Following the IPA wash, the print supports were removed and the samples were UV cured in a Formlabs UV Cure Station according to Formlabs guidelines.

**Figure 2:**
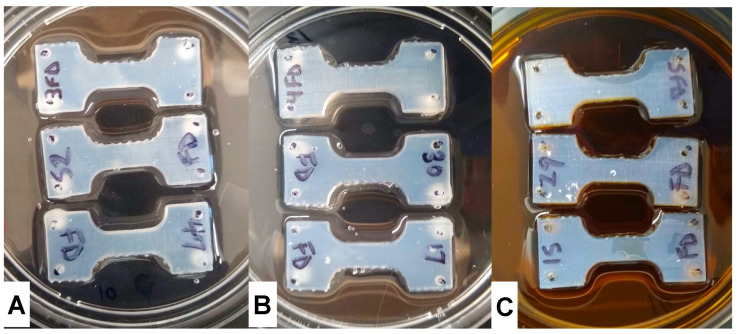
Micro-tensile sample liquid exposure at 37 °C. (**A**) Representative samples in water, (**B**) in PBS, pH 7.4, (**C**) in bile acid solution, pH 6.7. Samples were placed in 60 mm petri dishes with 30 mL of designated liquid and placed in incubator (N = 6 per liquid and time point). Note samples were not submerged in liquids.

Prior to liquid exposure the mass of each micro-tensile sample was recorded. One side of each sample was exposed to MilliQ water, phosphate buffered saline (PBS, pH 7.4), or bile acid solution (BAS) (Bile (Dried, Purified), HIMEDIA, VWR, Radnor, PA, USA 10% *m*/*m* in MilliQ water, pH 6.7) for 3 days or 7 days at 37 °C (Figure 2). After liquid exposure, samples were removed from the liquids, patted dry with a Kimwipe and their masses were recorded. 

Samples designated for imaging via scanning electron microscope (SEM) were dried for 3 h at 37 °C followed by sputter coating with a gold/palladium alloy. The resulting samples were imaged using a TESCAN VEGA 3 XMU VP-SEM (TESCAN USA, Warrendale, PA, USA) with a LaB_6_ emitter and utilizing a secondary electron detection.

Tensile tests to failure on the micro-tensile samples were performed within two hours of conclusion of the liquid exposure on a Mark-10 ESM303 at a stretch speed of 5 mm/s (*N* ≥ 6 per condition). Data were recorded at a rate of 10 samples per second.

Statistical analysis was performed via single factor analysis of variance (ANOVA). Stress–strain curves were obtained by plotting the averages for *N* ≥ 6 samples per condition.

### 2.3. EHBD Model Use in an In Vitro Biliary Stent Testing System

Plastic biliary stents ranging in length from 40 mm to 120 mm (Flexima, Boston Scientific, Marlborough, MA, USA) and SEM biliary stents ranging in length from 40 mm to 60 mm (WallFlex Uncovered, Boston Scientific, Marlborough, MA, USA) were deployed in the Elastic EHBD model with standard 8–10F catheters (N ≥ 6 per stent type). Once stents were deployed, the EHBD models were connected via barbed 1/8 inch or ¼ inch PVDF connectors (Masterflex Union Fittings, Cole-Parmer, Vernon Hills, IL, USA) and size 16 tubing (Masterflex PharMed BPT, Cole-Parmer, Vernon Hills, IL, USA) to a peristaltic pump (Cole-Parmer, Vernon Hills, IL, USA) in line with a pulse dampener (Cole-Parmer, Vernon Hills, IL, USA) and a reservoir of bile acid solution (BAS) (Bile (Dried, Purified), HIMEDIA, VWR, 10% *m*/*m* in MilliQ water, pH 6.7) in a 37 °C water bath. Bile acid solution was pumped through the stented Elastic EHBD model at flow rates from 0.5 mL/min to 5.0 mL/min [21].

## 3. Results

### 3.1. CAD EHBD Model

**Figure 3:**
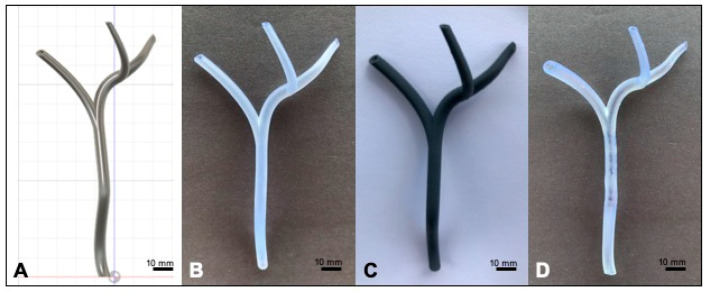
CAD extrahepatic bile ducts (EHBD) model and 3D printed EHBDs. (**A**) EHBD model in Fusion360; (**B**) EHBD model printed in Formlabs Durable resin with 0.5 mm duct walls; (**C**) EHBD model printed in Formlabs Flexible resin with 1 mm duct walls; (**D**) EHBD model printed in Formlabs Elastic resin with 0.75 mm duct walls.

Our CAD EHBD model had a uniform wall thickness throughout at 0.5 mm, 0.75 mm, or 1.0 mm. Designs were generated with three different wall thicknesses to compensate for the variations in achievable printing resolution across the three resins we tested. Figure 3A shows our EHBD CAD model with a wall thickness of 0.75 mm. The CBD and the CHD have inner diameters of 5.0 mm. The lengths of the CBD and CHD are 78.6 mm and 30.0 mm. The cystic duct has a diameter of 5.0 mm when it branches off of the common bile duct and tapered along its length of 4.0 cm to a diameter of 3.6 mm. The left and right hepatic duct narrowed from their junction with the common hepatic duct to 2.0 mm and 2.5 mm and are 2.9 cm and 3.8 cm in length, respectively. We oriented the cystic duct, CHD, LHD, and RHD so they bend and reach into all 3 geometric planes (Supplementary Files S1 and S2).

### 3.2. 3D Printed EHBD Models

Formlabs Durable, Flexible, and Elastic resins were selected for this study. Formlabs material datasheets indicated the maximum print resolution possible was 50 μm for Durable and Flexible resins and 100 μm for Elastic resin. These print resolutions were compatible with the three wall thicknesses we utilized in our CAD EHBD models.

Through a combination of following Formlabs PreForm guidelines for print orientation and trial and error, we determined that the EHBD model must be angled ~45° from the print platform (Figure S1, Supplementary File S3). Angles greater than 45° resulted in support scaffolding misprints and subsequent print errors of the EHBD model. Supports (touchpoint size 0.4 mm) were manually placed along the ducts to ensure adequate scaffolding was created by PreForm. We found spacing of the support touchpoints to be critical; if they were too close together, they often fused, producing a touchpoint that could not be detached without damaging the model.

Prints of the EHBD model at all wall thicknesses (WT) were successful in each resin but printing artifacts and difficulties with post-processing determined the wall thickness version evaluated for each resin. All of the 1 mm-walled EHBD models had no major printing or post-processing issues. We did find that the walls of the LHD and RHD in the printed Elastic EHBD would be thicker than in the CAD model as a consequence of the viscosity of the resin and laser scatter during printing. This caused a narrowing of the duct lumens and decreased flexibility of the ducts, neither of which were desirable.

To compensate for the resin viscosity and laser scatter (note: the Form2 and Form3 do heat the resins during printing to mitigate this issue), we printed Elastic EHBD models with 0.5 mm walls. However, minor jostling when these Elastic EHBD models with 0.5 mm walls were transferred from the printer to the wash station caused tears along the CBDs and CHDs. Ultimately, we found that 0.75 mm-walled EHBD CAD models printed in Elastic resin could endure handling during post-processing and had duct WT of 1 mm ± 0.1 mm (N ≥ 10). 

Figure 3 shows our EHBD models printed in Durable (Figure 3B, 1.0 mm WT), Flexible (Figure 3C, 1.0 mm WT), and Elastic (Figure 3D, 0.75 mm WT). Qualitatively, the Durable EHBD offered some visibility of duct contents but was stiff and brittle. The Flexible EHBD, as its name implies, was very flexible with moderate rebound capacity, although its opacity was a serious drawback for our intended application. Once UV-cured, the Elastic EHBD was resilient, pliable, and provided excellent visualization of the duct interiors. Per our goal of fabricating an EHBD model with tissue-like mechanical properties, we elected to move forward with mechanical testing of the Formlabs Elastic polymer only.

### 3.3. Mechanical Properties of Formlabs Elastic Polymer

**Figure 4:**
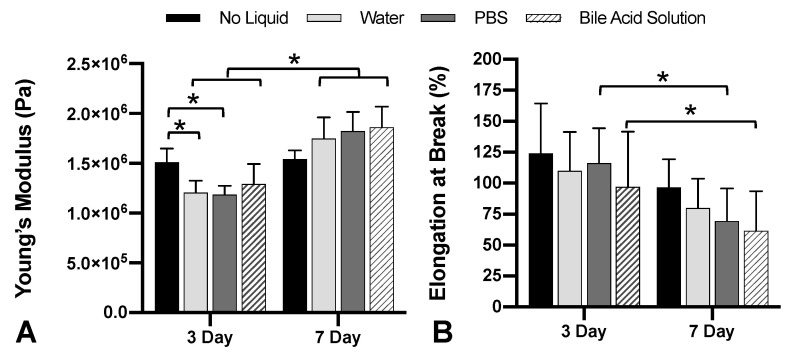
Mechanical properties of Formlabs Elastic polymer after liquid exposure at 37 °C. (**A**) Heat alone did not cause a change in Young’s modulus. Young’s modulus significantly decreases after 3 days exposed to heat and water or PBS (N = 6, * = *p* < 0.05) and increases after 7 days in all liquids (N = 6, * = *p* < 0.001). (**B**) Decreases in elongation at break, significant in PBS- and bile acid solution (BAS)-exposed samples (N = 6, * = *p* < 0.05), reflect a reduction in peak tensile strength.

We found our room temperature (RT), untreated Elastic samples to have a Young’s modulus of 1.71 MPa ± 0.169 MPa. Next, we exposed Elastic polymer samples to no liquid, water, PBS, or BAS at 37 °C for three or seven days. When samples were exposed to any of the liquids at 37 °C for three days, we saw significant decreases in the Young’s moduli relative to dry, 37 °C samples (Figure 4A; no liquid: 1.51 MPa ± 0.138 MPa, water: 1.21 MPa ± 0.119 MPa, PBS: 1.19 MPa ± 0.089 MPa, BAS: 1.29 MPa ± 0.202 MPa, see Figure S2A for stress–strain curves). 

Liquid-exposed sample weights for water, PBS and BAS increased by 0.63% ± 0.06%, 0.56% ± 0.06%, and 1.65% ± 0.08%, respectively, over 3 days at 37 °C. At 3 days exposure, we also found that all samples incubated showed significantly increased percent elongation at break, an indicator of increased tensile strength (Figure 4B; no liquid: 124.1% ± 40.1%, water: 110.1% ± 31.4%, PBS: 116.1% ± 28.3%, BAS: 97.1% ± 44.4%). 

After 7 days of liquid exposure at 37 °C we saw the Young’s Moduli return to values over 1.5 MPa (Figure 4A; no liquid: 1.54 MPa ± 0.088 MPa, water: 1.75 MPa ± 0.212 MPa, PBS: 1.82 MPa ± 0.194 MPa, BAS: 1.86 MPa ± 0.207 MPa, see Figure S2B for stress–strain curves). This increase in stiffness, a return to values similar to RT, untreated samples, occurred in spite of all of the samples absorbing additional liquid. The increase in weight was 1.33% ± 0.06% for water samples, 0.99% ± 0.08% for PBS samples, and 2.72% ± 0.07% for BAS samples (*p* < 0.0001). 

In parallel with the change in Young’s moduli, the percent elongations of our 7-day samples reverted to measurements similar to RT, untreated samples (Figure 4B; no liquid: 96.7% ± 22.7%, water: 79.8% ± 23.8%, PBS: 69.5% ± 26.3%, BAS: 61.6% ± 31.8%).

**Figure 5:**
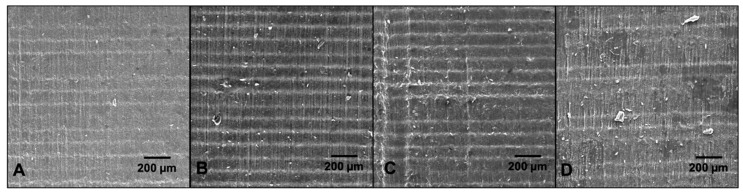
Surface characterization of Formlabs Elastic polymer after 7 day liquid exposure at 37 °C. Representative SEM images from (**A**) control, (**B**) water-exposed, (**C**) PBS-exposed, (**D**) BAS-exposed (N = 3 per condition).

SEM images of samples exposed to liquid for 7 days revealed minimal changes to the material surfaces (Figure 5). The 100 μm layer height is clearly evident in all samples; however, the layers are more distinct in the liquid-exposed samples (Figure 5B–D) likely due to the liquid absorption we observed. In Figure 5D residue from the dried BAS is visible but the integrity of the material surface appears unaffected.

### 3.4. Elastic EHBD Use in an In Vitro Biliary Stent Testing System

**Figure 6:**
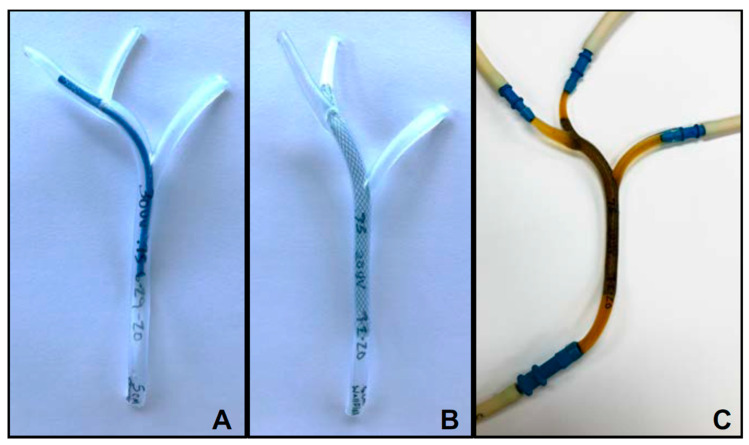
In vitro testing of biliary stents in the Elastic EHBD model. (**A**) Plastic biliary stent (7 F, 50 mm, Flexima) deployed into the right hepatic duct. (**B**) Uncovered SEM (8 mm × 60 mm Wallflex) deployed into the left hepatic duct. (**C**) Elastic EHBD with uncovered SEM deployed connected in line with a peristaltic pump, pulse dampener and bile acid solution reservoir. Bile acid solution was pumped through the model at 0.5 mL/min.

As a final evaluation step for our 3D printed Elastic EHBD, we deployed plastic or SEM biliary stents in various positions using standard 7–10 F catheters (See Supplementary Video S1). (Figure 6A shows a 5 cm, 7 F Flexima plastic stent deployed into the left hepatic duct. Figure 6B shows a 60 mm × 8 mm uncovered Wallflex SEM stent spanning from the left hepatic duct to the common bile duct. The optical clarity of the Elastic EHBD allowed for quick and easy placement of the stents in the desired locations. No damage resulted from typical catheter adjustments made during stent deployment. Once the stents were deployed the elasticity of the walls provided the radial forces required to hold the stent in place.

## 4. Discussion

### 4.1. Anatomic Accuracy of the FE EHBD Model

Bile, produced in the hepatocytes of the liver, is transported to the small intestine or to the gall bladder for storage by the bile ducts. The intrahepatic bile ducts originate in the distal lobes of the liver and converge into the extrahepatic bile ducts (EHBD). The EHBD connect the liver to the gall bladder and the small intestine. The branching pattern of the intrahepatic bile ducts is unique to each individual, but the orientation and size of the EHBDs are shared anatomical features across the majority of the population [22,23,24]. Imaging studies of the EHBD show healthy patients typically have a uniform EHBD wall thickness of 1.0 mm [18,19]. The wall thickness of our CAD EHBD model is an adjustable parameter; the CAD model used for our Elastic EHBD prints had a wall thickness of 0.75 mm to compensate for SLA printing artifacts in clear resins such as laser scatter. The increase in print thickness relative to the CAD dimensions is a consistent phenomenon; thus, once determined can be accounted for in the CAD file and reliability is still high for product tolerances. 

Regarding the duct lumens and lengths in our EHBD model, an inner diameter of 5.0 mm is slightly larger than the population average for the CBD found by Zuleta et al. but on par for the CHD [25]. The lengths of the CBD and CHD reflect the ranges of 60–80 mm and 10–75 mm for CBD and CHD respectively, as reported in previous studies [19,26]. The cystic duct of the CAD EHBD model has a diameter of 5.0 mm when it branches off of the common bile duct and is tapered along its length of 4.0 cm to a diameter of 3.6 mm [27]. Our selected lengths for the LHD and RHD (2.9 cm and 3.8 cm) reflect that the RHD is commonly slightly shorter than the LHD [28]. The diameters of the ducts can be easily modified and the orientation can also be readily manipulated should one need different duct dimensions to represent variations in patients’ biliary anatomy. 

### 4.2. Tissue-Like Mechanical Properties of the 3D Printed FE EHBD Model

Our aim was to identify which Formlabs resin could be used to reliably and accurately print our EHBD model. We intended the model to be anatomically accurate and possess similar mechanical properties to the EHBD in vivo; upon qualitative evaluation of EHBD prints in FE, FD, and FF, we concluded that FD and FF EHBD were unsuitable for our application. The FD EHBDs were dimensionally accurate but lacked flexibility. The FF EHBDs were completely opaque which would hinder accurate stent placement during experiments. 

We sought confirmation that the FE EHBD had similar mechanical properties to the EHBD in vivo and maintained those properties under the conditions required for in vitro testing of biliary stents, e.g., deployment or patency tests. Studies on the mechanical properties of the bile ducts are scarce [29]. In the early ‘90s, Jian and Wang [30] observed in dogs that the elastic modulus decreases moving into the liver from the common bile duct to the common hepatic duct to the left and right hepatic ducts. More recent work by Li [12] reported an elastic modulus of 1.26 kPa ± 0.07 kPa for human common bile duct. Based on the structural similarities to the small intestine, i.e., the mucosa of the small intestine and the EHBD are both comprised of a layer of smooth muscle and a layer of connective tissue, the mechanical properties of the small intestine mucosa can also serve as baselines. The elastic moduli of porcine and human small intestine are 1.03 MPa ± 0.57 MPa and 1.07 MPa, respectively [31,32]. We found our room temperature (RT), untreated Elastic samples to have a Young’s modulus of 1.71 MPa ± 0.169 MPa. While slightly higher than the reported gastrointestinal tissue Young’s moduli [31,32], this value is still representative of a highly elastic material.

Basic requirements for an in vitro stent testing system include that the system (1) holds a stent at body temperature (37 °C), (2) moves bile acid solution through the stent at low volumetric flow and low pressure, and (3) duct material does not rapidly fatigue under those conditions. In turn, we exposed Elastic polymer samples to no liquid, water, PBS, or BAS at 37 °C for three or seven days. After three days, we saw an initial decrease in Young’s modulus for FE and we speculate that this increase in elasticity, bringing the values even closer to those measured in ex vivo bile duct and small intestine specimens, can be attributed to the FE absorbing liquid. After seven days, all of the liquid-exposed samples showed decreases in elasticity. The ongoing exposure to 37 °C likely caused the decrease in elasticity; Formlabs Elastic resin datasheet notes that prolonged exposure to UV and/or high temperatures can affect the mechanical characteristics of prints made with Elastic resin. Fortunately, the change in Young’s moduli and percent elongation, though statistically significant, were not large. In addition, we do not anticipate a small decrease in tensile strength to adversely impact our intended application, so we maintain that the EHBD printed in Formlabs’ Elastic resin is highly suitable for use in an in vitro biliary stent testing system.

### 4.3. Comparison of In Vitro Biliary Stent Testing Systems

Previous in vitro biliary stent testing systems, while meeting the basic requirements described above for an in vitro stent testing system, have lacked the combination of features found in our FE EHBD model. Bang et al. [8] and Kwon et al. [33] utilized lengths of silicone tubing connected in parallel to a pump system. Silicone tubing can approximate the length and diameter of the CBD and CHD, but it does not reflect the branching of the biliary tree or the mechanical properties of biliary tissue. In 2019, Huang published work with a 3D printed EHBD model based on an MRI of the bile ducts [10]. Admittedly, their model is singularly anatomically accurate. However, the model appeared to be printed in a rigid material to achieve the resolution needed to reproduce the MRI-derived model. Thus, stents deployed in their EHBD model will not experience external forces similar to those generated by the bile ducts in vivo. In contrast with these systems, when stents are deployed in our Elastic EHBD model (Figure 5A,B) and then connected in line with a peristaltic pump and pulse dampener (Figure 5C), the stents experience the physical orientation and combination of forces (pressure, friction, and fluid shear) similar to those that they would experience in vivo. 

## 5. Conclusions

An ideal in vitro biliary stent testing system would be a bioreactor that provides an environment that is both anatomically accurate and physiologically accurate. Tissue engineered EHBD could potentially meet those requirements; however, fabrication of tissue engineered BD, thus far, is time and consumable intensive. Many tissue engineering protocols rely on multi-day culture timelines and numerous reagents to evaluate products [34,35,36,37]. Reactors housing organs or vessels require calibrated perfusion [38,39,40]. Scale-up and product transport remain challenges for tissue engineered products [41,42,43]. The hours of labor, the facilities, and the supplies needed to grow the EHBD required to test a statistically significant number of stents is, at this time, cost prohibitive for biliary stent product development applications [44,45].

Our results demonstrate that our FE EHBD model is anatomically accurate with similar mechanical properties to biliary and intestinal tissues. We also show, from a manufacturing perspective, that we are able to reliably, and precisely fabricate our EHBD model with commercially available equipment and materials. These features make our EHBD model a cost-effective means to generate data on stent mechanics and/or stent patency with an in vitro biliary stent testing system. 

In the future, we plan to address ways in which we can improve the anatomical and physiological accuracy of our Formlabs Elastic EHBD model. Formlabs does not indicate biocompatibility of FE but it does report that FE is autoclavable with minimal impact to mechanical properties [46]. This will facilitate our testing of the biocompatibility of FE. Results from cytotoxicity tests with FE will dictate future plans for experiments to assess FE’s capacity to support cell growth. Another area of focus will be generating a library of CAD models that reflects the various orientations and dimensions of the EHBD observed in vivo among patients and modifying the lumen of the cystic duct to include the baffle-like spiral valves of Heister. We will also conduct additional time course studies to evaluate any further effects exposure to bile acid solution and body temperature may have on the mechanical properties of Formlabs Elastic material.

## Supplementary Materials

The following are available online at https://www.mdpi.com/1996-1944/13/21/4788/s1. Figure S1: EHBD orientation on print platform. Samples were manually positioned at ~ 45° angle to the print platform to promote resin drainage between layers and minimize polymerization due to laser scatter. This angle also ensured the height of the support scaffolding would not result in misprints, Figure S2: Transient increase in FE elasticity upon exposure to liquids. (A) Stress–strain analysis after 3 day liquid exposure. All samples exposed to liquids experienced a significant decrease in elastic modulus (see Figure 4). (B) Stress–strain analysis after 7 day liquid exposure. All increases in elasticity seen at three days were reversed after one week. Stress–strain curves represent the average values taken from N = 6 samples. See Section 3.3 for statistical analysis of the change in Young’s modulus, Video S1: Biliary stent deployment in EHBD model demo video, File S1. EHBD model Fusion360 file, File S2: EHBD model .STL file, File S3: EHBD model .form file (for printing with Formlabs SLA printers).
