# Supplementary material for: 3D Printed Model of Extrahepatic Biliary Ducts for Biliary Stent Testing
Source: Materials (Basel). 2020 Oct 27;13(21):4788. doi: 10.3390/ma13214788 (PMC7663029; doi:10.3390/ma13214788)
Supplement: Supplementary file 1 [file materials-13-04788-s001.pdf]

materials

Supplement Materials

3D Printed Model of Extrahepatic Biliary Ducts for Biliary Stent Testing

Joanna Thomas [1,*], Sagar Patel [1], Leia Troop [1], Robyn Guru [1], Nicholas Faist [1], Brian J. Bellott [2] and Bethany A. Esterlen [2]

[1] Biomedical Engineering Department, Mercer University, Macon, GA 31207, USA; patel_SG@mercer.edu (S.P.); leia.danielle.troop@live.mercer.edu (L.T.); robyn.guru@live.mercer.edu (R.G.); robyn.guru@live.mercer.edu, Nicholas.ryan.faist@live.mercer.edu (N.F.)

[2] Chemistry Department, Western Illinois University, Macomb, IL 61455, USA; b-bellott@wiu.edu (B.J.B.); Ba-esterlen@wiu.edu (B.A.E.)

* Correspondence: Thomas_JL@mercer.edu

Received: 27 September 2020; Accepted: 23 October 2020; Published: date

Supplementary Videos

Video S1. Biliary stent deployment in EHBD model demo video

Supplementary Files

File S1. EHBD model Fusion360 file

File S2. EHBD model .STL file

File S3. EHBD model .form file (for printing with Formlabs SLA printers)

Supplementary Figures

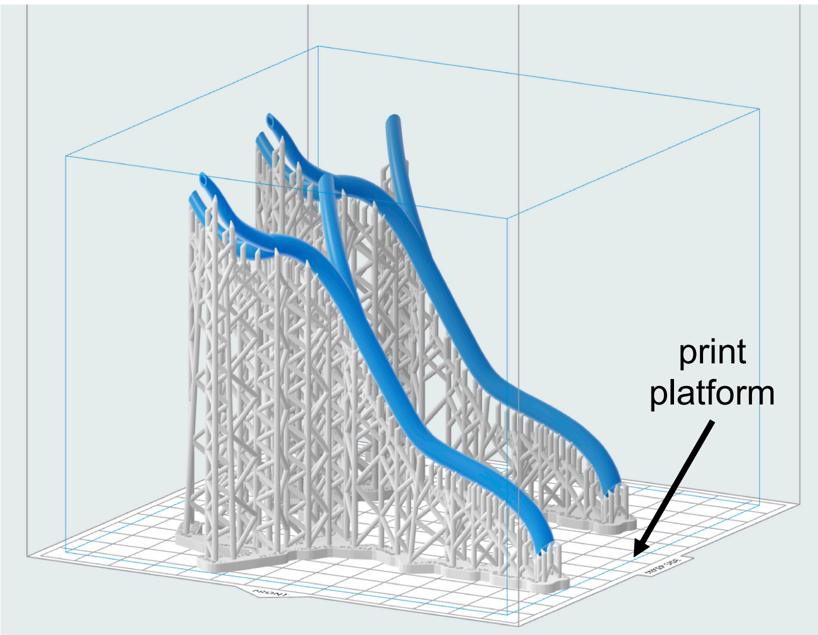

Figure S1. Extrahepatic bile ducts (EHBD) orientation on print platform. Samples were manually positioned at ~ 45° angle to the print platform to promote resin drainage between layers and minimize polymerization due to laser scatter. This angle also ensured the height of the support scaffolding would not result in misprints.

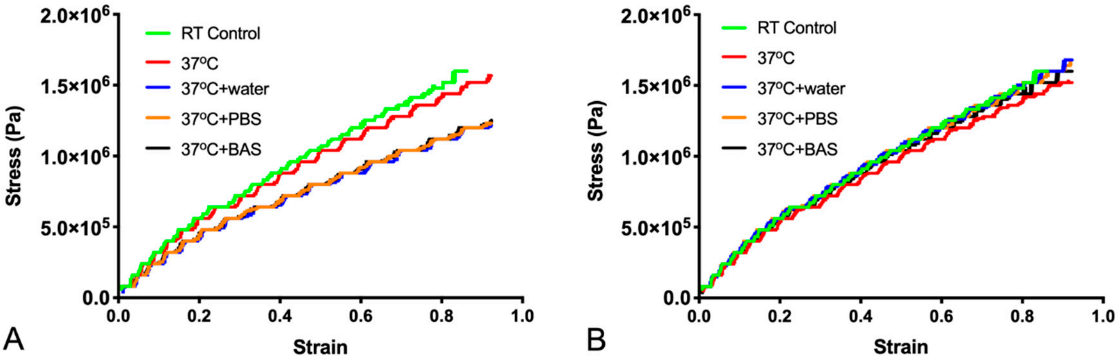

Figure S2. Transient increase in FE elasticity upon exposure to liquids. (**A**) Stress–strain analysis after 3 day liquid exposure. All samples exposed to liquids experienced a significant decrease in elastic modulus (see Figure 4). (**B**) Stress–strain analysis after 7 day liquid exposure. All increases in elasticity seen at three days were reversed after one week. Stress–strain curves represent the average values taken from N = 6 samples. See Section 3.3 for statistical analysis of the change in Young's modulus.

Supplementary Methods: Optimized EHBD Model Post-Processing

Once the EHBD 3D printing is complete (~20 hrs), it undergoes the following post-processing:

- Dip right hepatic duct (RHD), left hepatic duct (LHD), and cystic duct ends in >95% isopropanol (IPA).
- Wearing gloves, carefully squeeze the excess resin from the duct ends with your fingers.
- With the EHBD print still attached to print bed, wash in >95% IPA for 30 min in a Formlabs Wash station.
- When wash is complete, gently repeat removing any excess resin from the duct ends.
- Wash 10 min in >95% IPA.
- While washing, pre-heat the Formlabs UV cure station to 60 °C.
- While the EHBD is still wet from IPA wash, very carefully remove from the print bed by sliding your fingers along the ducts and gently squeezing the supports to detach them. It is critical that the EHBD is still wet with IPA and remains suspended from the print bed as you detach the supports. Inverting the print bed can cause excess strain from the support structure on the EHBD. The strain leads to tears in the ducts.
- Immediately place the EHBD in the ultraviolet (UV) cure station and UV cure for 20 min at 60 °C.
- Remove the EHBD from UV cure station immediately when cure cycle is complete.
- Handle EHBD carefully until cool.
